# GEOGRAPHIC DISPARITIES IN ALZHEIMER’S DISEASE AND RELATED DEMENTIAS USING BAYESIAN MULTISTATE LIFE TABLE METHODS

**DOI:** 10.1093/geroni/igae098.0691

**Published:** 2024-12-31

**Authors:** Jason Wong, Emma Zang

**Affiliations:** Yale University, New Haven, Connecticut, United States; Yale University, New Haven, Connecticut, United States

## Abstract

Research has documented persistent regional disparities in the risk of Alzheimer’s disease and related dementias (ADRD) in the United States based on individuals’ current residence. However, it remains unclear how childhood region and current region jointly shape later ADRD outcomes. This obscurity conceals significant variations among older adults who reside in the same current region. Using data from the 1998-2020 waves of the Health and Retirement Study, we employ an innovative Bayesian multistate life table method to estimate how ADRD-related life expectancies vary across combinations of birth regions and current regions. Our results show that ADRD-related life expectancies are more strongly associated with regions of birth than with current regions of residence. Especially, at age 50, individuals born in the South, regardless of current residence, were expected to have shorter years (Men: 13.8-15.2 years; Women: 17.3-18.4 years) of life living without ADRD and longer years (Men: 2.7-3.0 years; Women: 3.6-3.9 years) of life with dementia, compared to those born in other regions. Our findings offer new evidence of the Southern disadvantage of ADRD by demonstrating that growing up in the South during the critical period of childhood may have long-lasting impacts on cognitive functioning later in life.

